# Host Reticulocyte Redox Attenuation Creates a Protective Niche for Artemisinin Tolerance in *Plasmodium falciparum*

**DOI:** 10.1093/infdis/jiag290

**Published:** 2026-06-16

**Authors:** Christeen Davis, Lakshmi V S, Sreenath Meloth, Renugah Naidu, Trang T T Chu, Rema Sreejith, Kamalakannan Vijayan, Manish Kumar, Dhanasekaran Shanmugam, Rajesh Chandramohanadas

**Affiliations:** Division of Pathogen Biology, Biotechnology Research and Innovation Council (BRIC)-Rajiv Gandhi Centre for Biotechnology (RGCB), Thiruvananthapuram, Kerala, India; Manipal Academy of Higher Education, Manipal, Karnataka, India; Division of Pathogen Biology, Biotechnology Research and Innovation Council (BRIC)-Rajiv Gandhi Centre for Biotechnology (RGCB), Thiruvananthapuram, Kerala, India; Regional Centre for Biotechnology, Faridabad, Haryana, India; Division of Pathogen Biology, Biotechnology Research and Innovation Council (BRIC)-Rajiv Gandhi Centre for Biotechnology (RGCB), Thiruvananthapuram, Kerala, India; Pillar of Engineering Product Development, Singapore University of Technology and Design, Singapore, Singapore; Pillar of Engineering Product Development, Singapore University of Technology and Design, Singapore, Singapore; Department of Obstetrics and Gynaecology, Cosmopolitan Hospital, Thiruvananthapuram, Kerala, India; School of Biology, Indian Institute for Science Education and Research, Thiruvananthapuram, Kerala, India; School of Biology, Indian Institute for Science Education and Research, Thiruvananthapuram, Kerala, India; Biochemical Sciences Division, Council of Scientific and Industrial Research (CSIR)- National Chemical Laboratory (NCL), Pune, Maharashtra, India; Division of Pathogen Biology, Biotechnology Research and Innovation Council (BRIC)-Rajiv Gandhi Centre for Biotechnology (RGCB), Thiruvananthapuram, Kerala, India; Regional Centre for Biotechnology, Faridabad, Haryana, India; Pillar of Engineering Product Development, Singapore University of Technology and Design, Singapore, Singapore

**Keywords:** *Plasmodium falciparum*, artemisinin tolerance, reticulocyte tropism, oxidative stress, redox attenuation, host cell-mediated drug response

## Abstract

**Background:**

Artemisinin-based combination therapies are the cornerstone of *Plasmodium falciparum* malaria treatment; however, delayed parasite clearance and treatment failure occur even in the absence of established parasite genetic resistance. While parasite-intrinsic resistance mechanisms have been extensively studied, the contribution of host erythroid heterogeneity to antimalarial drug response remains poorly defined.

**Methods:**

Highly purified human CD71^+^ reticulocytes and mature erythrocytes were used to assess parasite invasion, intraerythrocytic development, and drug susceptibility. Integrated metabolomic and proteomic analyses characterized host cell environments. Parasite responses to artemisinin derivatives and antimalarials with distinct mechanisms of action were systematically evaluated.

**Results:**

*P. falciparum* preferentially invaded reticulocytes and exhibited accelerated asexual development within these cells. Multiomics analyses demonstrated that reticulocytes provide a metabolically enriched, antioxidant-modulated intracellular environment, marked by increased nucleotide precursors, enhanced glutathione metabolism, and elevated levels of reactive oxygen species-detoxifying enzymes. Parasites developing within reticulocytes showed significantly reduced susceptibility to artemisinin and dihydroartemisinin, as well as to other redox-active antimalarials, while maintaining comparable sensitivity to drugs with nonoxidative mechanisms. Reticulocyte-resident parasites also demonstrated enhanced survival and recrudescence following artemisinin exposure.

**Conclusions:**

Erythroid maturation state is a key host determinant of antimalarial drug efficacy. Reticulocytes form a redox-protected intracellular niche that attenuates drug-induced oxidative stress and promotes parasite persistence, consistent with a host cell-mediated tolerance phenotype. These findings provide a mechanistic framework for variability in treatment outcomes associated with reticulocytosis and anemia.

## Teaser

The maturation state of host erythroid cells shapes intracellular pathogen responses to oxidative stress and drug exposure. Metabolically active reticulocytes provide a redox-modulated niche that reduces drug efficacy and promotes survival, highlighting host cell physiology as a determinant of therapeutic outcomes.

## Graphical Abstract

**Graphical Abstract:**
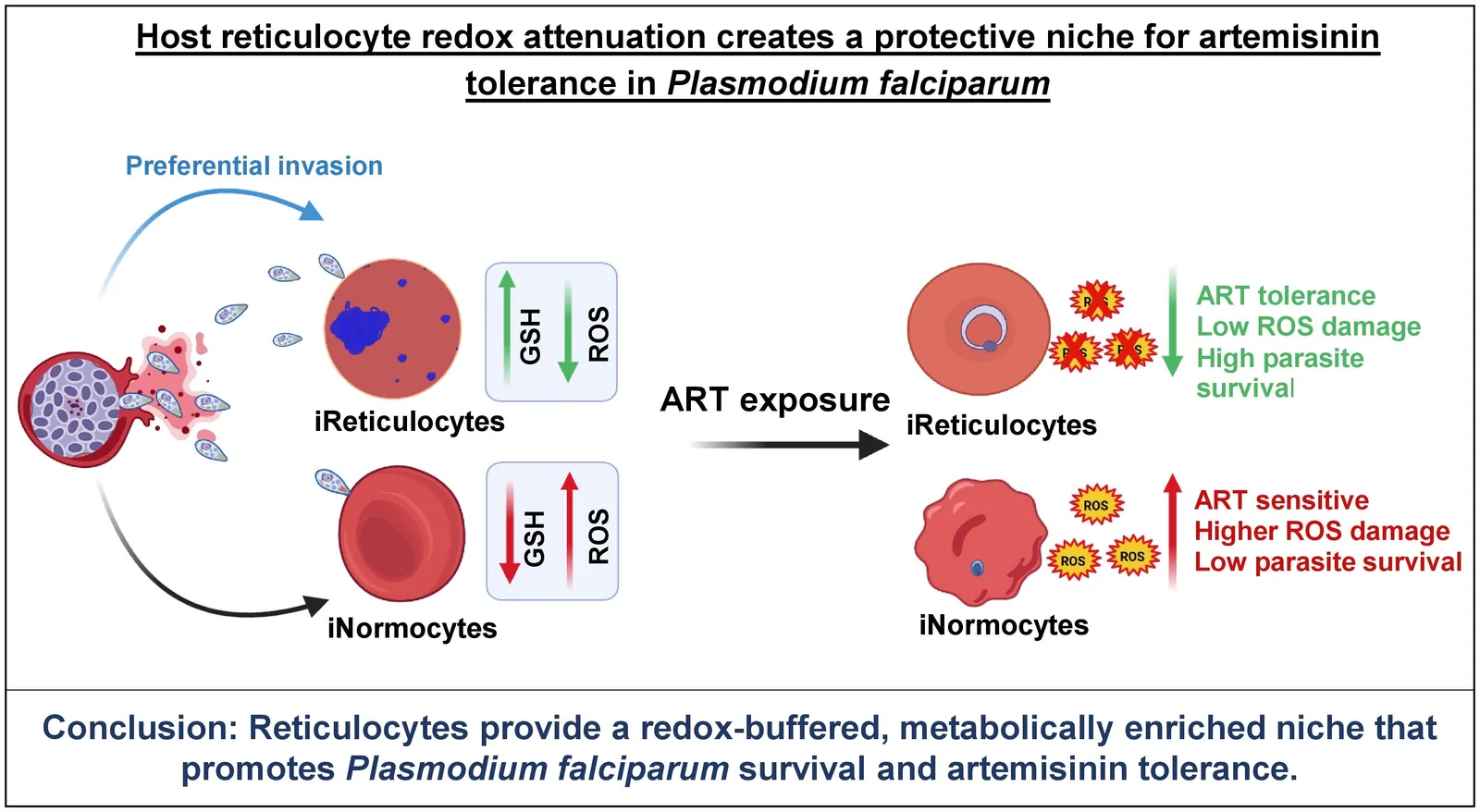

Intraerythrocytic development of *Plasmodium* determines parasite burden and disease severity. A defining feature of blood-stage malaria is parasite tropism for erythrocytes of distinct maturational states. While *Plasmodium vivax* and *Plasmodium ovale* exhibit strict preference for immature red blood cells (reticulocytes), *Plasmodium malariae* favors senescent erythrocytes [1–6]. *P. falciparum* is capable of invading erythrocytes across the full age spectrum, although with a documented bias toward younger cells [7, 8]. In rodent malaria, parasites such as *Plasmodium berghei* show extreme reticulocyte restriction, whereas zoonotic species, including *Plasmodium cynomolgi* and *Plasmodium knowlesi,* display host-dependent shifts in erythrocyte preference [9–12]. These invasion patterns are shaped by both receptor availability (eg, CD71, CR1, DARC) and the biochemical properties of erythroid subpopulations [1, 3, 6], with consequences for parasite replication, transmission, immune evasion, and clinical outcomes [9, 10, 13–16].

A central determinant of blood-stage development is the parasite's reliance on host metabolism. *P. falciparum* lacks complete biosynthetic pathways for purines, lipids, and several cofactors, and therefore depends heavily on host-derived metabolites [17, 18]. Reticulocytes, unlike mature normocytes, remain metabolically active and contain abundant nucleotide precursors, amino acids, and redox intermediates [19–21]. Parasites further remodel host glycolysis to meet energetic demands, consuming the majority of available glucose in infected cells [22, 23]. Oxidative stress generated during hemoglobin digestion is buffered by host glutathione-dependent antioxidant systems, and infected erythrocytes exhibit marked depletion of reduced glutathione and accumulation of oxidized species [24–27]. Consistent with this reliance, parasites defective in pyrimidine biosynthesis or heme detoxification can survive only within reticulocytes, underscoring the compensatory metabolic support provided by immature erythroid cells [19, 28].

These advantages arise from fundamental differences between reticulocytes and normocytes. Reticulocytes retain residual organelles, including mitochondria and ribosomes, supporting elevated biosynthetic and metabolic activity [29]. They are larger, less deformable, and enriched for invasion-associated host surface receptors [2, 3, 6, 30, 31]. In contrast, normocytes are metabolically quiescent and function primarily as oxygen carriers. Together, these features create a uniquely permissive intracellular environment for parasite invasion, growth, and stress tolerance. Intracellular pathogens frequently exploit host antioxidant systems to withstand oxidative stress arising from metabolism, immune attack, or nutrient digestion. *P. falciparum* parasites have been shown to import host antioxidant enzymes, including peroxiredoxins, which contribute to intracellular redox homeostasis [32, 33]. Similar host-derived redox modulation strategies are observed in other intracellular pathogens, highlighting the importance of host cell redox state in shaping pathogen survival and stress tolerance [34–36].

Here, we provide systematic experimental evidence that *P. falciparum* preferentially invades CD71^+^ reticulocytes and undergoes accelerated intraerythrocytic development within these cells. We show that the metabolically enriched, antioxidant-buffered reticulocyte environment counteracts artemisinin-induced oxidative stress, resulting in reduced drug susceptibility and enhanced parasite survival. Importantly, this phenotype reflects host cell-mediated drug tolerance rather than parasite-intrinsic resistance. Given that reticulocytosis is a defining feature of pediatric malaria and anemia, our findings establish erythroid maturation state as a critical host determinant of antimalarial drug efficacy.

## METHODS

### Ethics Statement

The protocol for blood collection from healthy adults was approved by the Institutional Human Ethics Committee (IHEC/1/2022_1/02) at the Biotechnology Research and Innovation Council (BRIC) -Rajiv Gandhi Centre for Biotechnology. Umbilical cord blood was collected from the Cosmopolitan Hospital, Thiruvananthapuram, from volunteers after normal term pregnancies, according to the approved protocols. This study utilized reticulocytes from 30 cord blood samples collected over a 3-year period. *P. falciparum* culture and blood handling protocols were approved by the Institutional Biosafety Committee.

### Blood Collection and Reticulocyte Enrichment

O^+^ blood collected into EDTA vacutainers was pelleted at 1000*g* for 5 minutes to collect erythrocytes. It was washed three times in Roswell Park Memorial Institute 1640 medium to remove any white blood cells, suspended in media containing gentamicin and maintained at 4°C for up to 2 weeks. For reticulocyte isolation (CD71^+^), blood was incubated with anti-CD71 antibody-coated magnetic beads and isolated on a magnetic separator (Miltenyi Biotec) [37]. On average, 1 mL of packed cord blood yielded 25 µL reticulocytes, which were used at 1% hematocrit for *P. falciparum* infection experiments.

### Parasite Culturing

All experiments were performed using *P. falciparum* 3D7 strain, unless stated otherwise. Parasites were maintained in O^+^ blood at 2% hematocrit in RPMI 1640 (10.4 g/L) supplemented with AlbuMAX I (0.5% wt/vol), HEPES (25 mM), hypoxanthine (100 µM), gentamicin (10 µg/mL), and sodium bicarbonate (177 mM). Parasite cultures were maintained in an incubator with 5% CO_2_ [38]. Parasitemia was monitored through microscopic examination of Giemsa-stained smears. Parasitemia was estimated by counting 1000 cells per smear. Parasite synchrony was achieved by treatment with 5% D-sorbitol (rings) or magnetic selection (schizonts) [38].

### Parasite Invasion and Multiplication Assays

Schizont-stage parasites were mixed with enriched reticulocytes and normocytes to make up an initial parasitemia of 0.7%–1% at 1% hematocrit in 96-well plate format. Ring-stage infections (18–20 hours postinfection [hpi]) in the subsequent cycle were counted from Giemsa-stained smears. The proportion of monoinfected and multi-infected erythrocytes was quantified from the Giemsa-stained thin films. Infected erythrocytes with more than 1 ring-stage parasite were counted as multi-infected. A total of 1000 infected erythrocytes were counted for this estimation. Student *t* test was used to confirm statistical significance.

To determine the invasion preferences, reticulocytes were stained with carboxyfluorescein succinimidyl ester (CFSE) (excitation, 492 nm; emission, 517 nm) while the normocytes were not stained (CD71^−^, CFSE^−^). Different ratios of reticulocytes versus normocytes were prepared and seeded along with magnet-isolated schizonts (0.7%–1% initial parasitemia and 1% hematocrit). At late-ring stages (approximately 20 hpi), samples were fixed with 0.125% glutaraldehyde in 1 × phosphate-buffered saline and stained using Hoechst 33342 (excitation, 361 nm; emission, 486 nm) for flow cytometry (BD ARIA III flow cytometer). The fraction of infected reticulocytes for each combination was estimated to derive preference patterns. Flow plots were analyzed using FlowJo V10.

To estimate merozoite number, parasites seeded into red blood cells (RBCs) of choice were allowed to progress until schizonts (approximately 45 hpi). Samples were then stained with Hoechst 33342 for 5 minutes and smeared on to glass slides for counting using a Nikon Eclipse Ni-E microscope. Merozoites from 15 schizonts per sample were counted to determine the parasite replication rate. For estimating the duration of parasite development, the number of schizonts from infected RBCs was tracked from 42 hpi for the next 10 hours through flow cytometry and microscopy. The time point at which the schizont population was reduced to 50% was considered as the egress time.

### Determination of Drug Sensitivity

Comparative drug tolerances of *P. falciparum* inside the reticulocytes and normocytes were estimated using ring-to-ring assays over a 48-hour period. Artemisinin, dihydroartemisinin, cycloheximide, pyrimethamine, atovaquone, and E64d were chosen as inhibitors with a broad spectrum of activities. To ensure consistency, all experiments were conducted within a 48-hour period. Parasitemia was estimated from Giemsa-stained smears and validated with flow cytometry after Hoechst staining, with unstained samples serving as controls (Supplementary Figure 8). Fifty percent maximum inhibitory concentration (IC_50_) was estimated by nonlinear regression (normalized) model using GraphPad Prism version 10.

### Resurgence and Ring-Survival Assays

Synchronized parasites grown in reticulocytes and normocytes were treated with different concentrations of artemisinin for 48 hours (ring to ring). The drug was removed by washing and parasites were allowed to progress for the next 10 days. No additional erythrocytes were added to the samples after artemisinin exposure to reduce any heterogeneity owing to reticulocyte maturation. Parasitemia was monitored using Giemsa-stained smears. Resurgence was defined as reaching 10% parasitemia. Untreated controls and parasites exposed to low artemisinin concentrations resurged by day 2. Parasite survival was estimated using Mantel-Cox rank analysis.

For ring survival assay, cultures were treated with varying artemisinin concentrations at 0–3 hpi for 6 hours at 1% hematocrit and 2%–3% initial parasitemia. Parasitemia was estimated after 66 hours to estimate artemisinin tolerance compared to corresponding untreated controls [39]. No additional erythrocytes were added to the culture to reduce the heterogenicity of erythrocytes. Parasites were cultured for 1 cycle in reticulocytes, transferred to normocytes, and ring-to-ring assay against artemisinin was performed, to ascertain reversibility of increased artemisinin tolerance.

### Mass Spectrometry

Detailed methodology followed for mass spectrometry is provided in the Supplementary Methods.

### Estimation of Reactive Oxygen Species

Reactive oxygen species (ROS) status of RBCs (1% hematocrit maintained in 100 µL in complete malaria culture medium) was estimated after challenging with tert-butyl hydroperoxide (TBHP), for 1 hour at 37°C. Samples were prestained with 5 µM 6-chloromethyl-2′,7′-dichlorodihydrofluorescein diacetae (CM-H_2_DCFDA) and the ROS generated was measured through flow cytometry. Median fluorescent intensities were used to ascertain the ROS tolerance [40, 41].

### Statistical Analysis

All experiments were performed with a minimum of 3 biological replicates. Unless otherwise mentioned, statistical significance was assessed using 2-tailed unpaired *t* test.

## RESULTS

### P. *falciparum* Preferentially Invades Immature CD71^+^ Reticulocytes

*Plasmodium* spp, with the exception of *P. malariae*, preferentially infect nascent human RBCs. Although *P. falciparum* exhibits a measurable bias toward younger erythrocytes [7], direct experimental evidence for a specific preference for reticulocytes remains limited. To systematically address this, we purified reticulocytes (CD71^+^) and normocytes (CD71^−^) by magnetic selection, achieving > 95% purity for infection assays. Although the reticulocyte population spanned all maturation stages, the majority were stages II–IV (Figure 1*A* and Supplementary Figure 1*A*).

**Figure 1.**
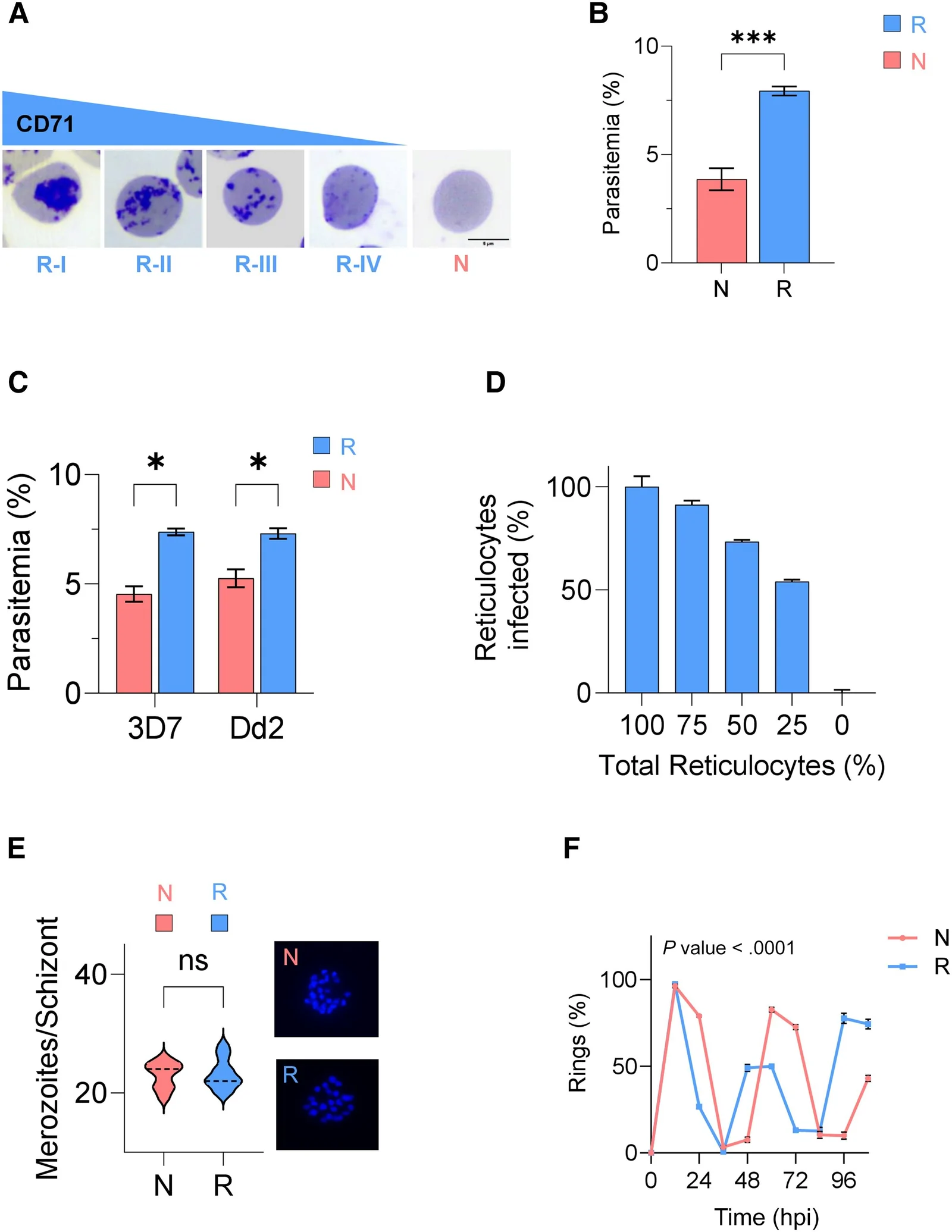
Preferential invasion and accelerated development of *Plasmodium falciparum* in human CD71^+^ reticulocytes. *A*, New methylene blue-stained images showing reticulocyte maturation stages (R-I to R-IV; CD71^+^) and normocytes (CD71^−^) purified from umbilical cord blood. *B*, Invasion efficiency of *P. falciparum* (strain 3D7) into reticulocytes and normocytes purified from peripheral blood, showing approximately 2-fold higher invasion into reticulocytes (mean ± SD, n = 3). Statistical significance was assessed using an unpaired *t* test. *C*, Comparative invasion profiles of *P. falciparum* strains 3D7 and Dd2 into cord blood-derived reticulocytes and normocytes, demonstrating a clear preference for reticulocytes (mean ± SD, n = 3). Statistical significance was assessed using an unpaired *t* test. *D*, Competition assay using CFSE-labeled reticulocytes and unlabeled normocytes mixed at defined ratios. The proportion of infected CFSE^+^ cells was quantified as a fraction of total parasitized red blood cells. When reticulocytes comprised 25% of the host population, nearly 50% of infections occurred in these cells, indicating parasite preference for immature blood cells. *E*, Parasite multiplication rate, measured by merozoite yield per schizont, was comparable irrespective of host cell types (unpaired *t* test, *P* ≥ .05), indicating that the reticulocyte environment influences developmental kinetics but not replication output. Merozoite counts were estimated from 15 segmented schizonts per sample. Representative images of segmented schizonts from reticulocytes (R, bottom) and normocytes (N, top) are shown. *F*, Line graph showing the abundance of ring-stage parasites at different time points to track parasite progression in reticulocytes and normocytes. Parasite development proceeded faster in reticulocytes than in normocytes, with the intraerythrocytic cycle completed approximately 4 hours earlier. The values are represented as mean ± SD from 3 independent observations. Statistical significance was determined using 2-way repeated measures ANOVA, where ns indicates *P* ≥ .05, *P ≤ .05 (significant), ***P ≤ .001. Abbreviations: CFSE, carboxyfluorescein succinimidyl ester; hpi, hours postinfection; N, normocyte; ns, not significant; R, reticulocyte.

We first compared invasion efficiency in reticulocytes and normocytes derived from peripheral blood. Parasites showed significantly higher invasion in reticulocytes, reflected by an increased population of newly formed rings (Figure 1*B*). This preference was independently validated using umbilical cord blood-derived reticulocytes for both 3D7 and Dd2 strains (Figure 1*C*). Given the higher reticulocyte content of cord blood (approximately 8% vs approximately 1.5% in peripheral blood), subsequent experiments used cord blood reticulocytes.

To examine the effect of reticulocytemia on invasion, reticulocytes were mixed with normocytes to generate cultures containing 0%, 25%, 50%, or > 95% reticulocytes. Increasing reticulocyte proportions led to a corresponding rise in total parasitemia (Supplementary Figure 2), consistent with preferential invasion. We further assessed invasion bias using a flow cytometry-based competition assay in which purified reticulocytes were CFSE-labeled (CD71^+^, CFSE^+^) and mixed with unlabeled normocytes (CD71^−^, CFSE^−^). Across all mixtures, invasion events were disproportionately enriched in reticulocytes (Figure 1*D*). Notably, in cultures containing only 25% reticulocytes, more than 50% of invasion occurred in reticulocytes, while > 80% of invasion was confined to reticulocytes in 50% reticulocyte cultures, indicating parasite preference toward immature erythroid cells. The gating strategy is shown in Supplementary Figure 3.

Preferential invasion did not result in increased multi-infected erythrocytes (Supplementary Figure 4*A*). To determine whether prior host cell residency influenced invasion behavior, parasites that had completed 1 developmental cycle in reticulocytes or normocytes were allowed to invade either cell type. Invasion into reticulocytes remained consistently higher irrespective of the erythrocyte population in which parasites had previously developed (Supplementary Figure 4*B*), confirming that invasion preference is determined by the age of the target erythrocyte.

Given the metabolic richness of reticulocytes, we next examined parasite development within these cells. Microscopic analysis of Giemsa-stained smears across multiple cycles revealed comparable parasite multiplication rates in reticulocytes and normocytes, as indicated by similar numbers of merozoites per schizont (Figure 1*E*). However, parasite development was accelerated in reticulocytes, with intraerythrocytic stages progressing approximately 4 hours ahead of those in normocytes (Figure 1*F*). This was corroborated by mean egress time measurements, which showed that approximately 50% of parasites completed egress approximately 4 hours earlier when developing in reticulocytes (Supplementary Figure 5*A* and 5*B*).

### Metabolomic Profiling Reveals a Nutrient- and Antioxidant-Rich Reticulocyte Niche

To define metabolic features underlying preferential invasion and accelerated development, we performed comparative metabolomic profiling of reticulocytes and normocytes. Reticulocytes displayed greater abundance and diversity of metabolites, particularly those associated with nucleotide metabolism, amino acid biosynthesis, redox regulation, and energy homeostasis (Figure 2*A* and 2*B*). Of 8061 metabolites identified, 2922 differed significantly between reticulocytes and normocytes (*P* < .05, log_2_ fold change > 1) and clustered distinctly by cell type (Figure 2*C*).

**Figure 2.**
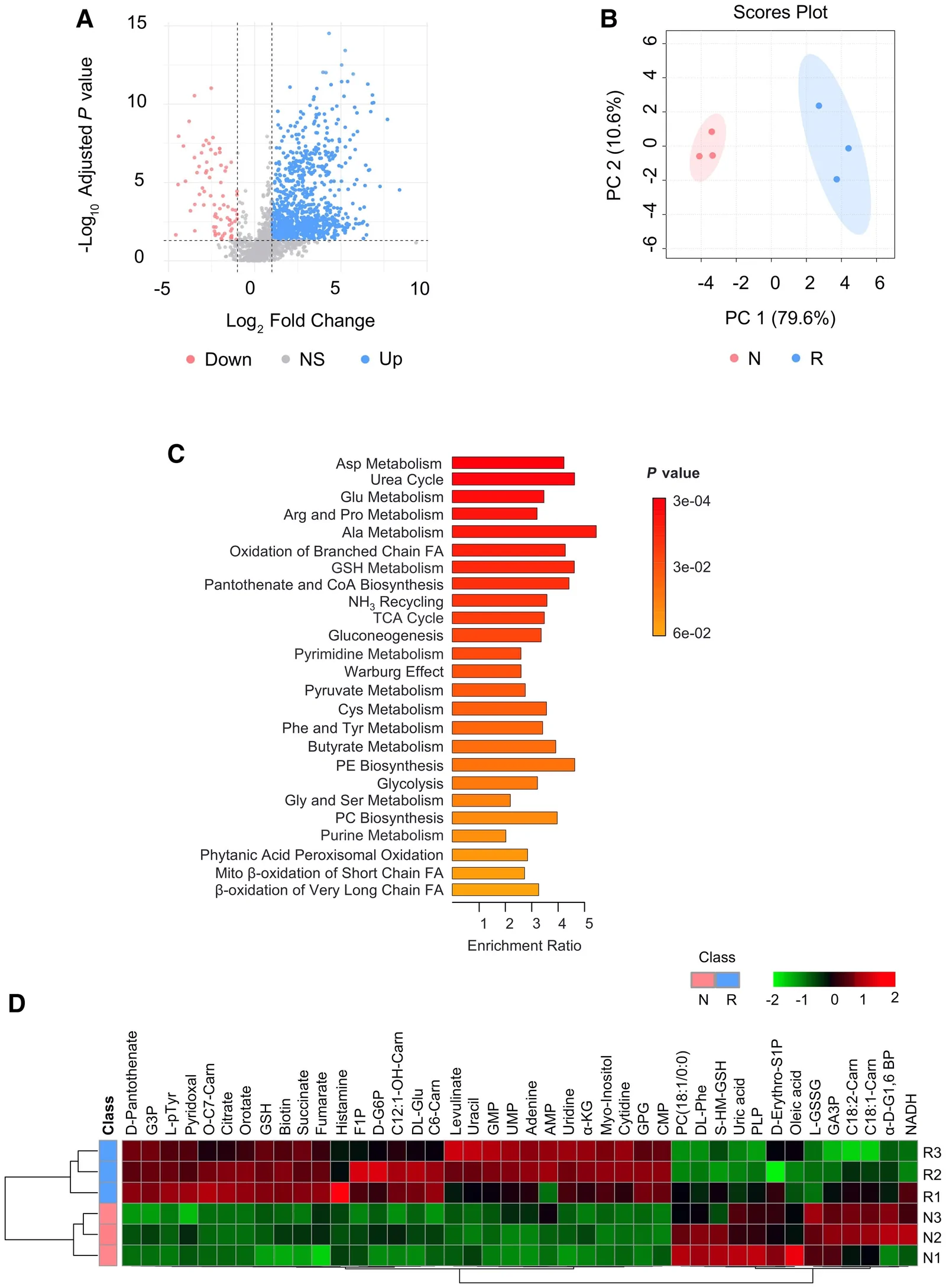
Metabolomic profiling of human reticulocytes and normocytes reveals distinct metabolic plasticity and enhanced redox homeostasis. *A*, Volcano plot comparing metabolite profiles of healthy reticulocytes and mature normocytes. Metabolites with -log_10_ (*P* > 1.3) and log_2_ fold change > 1 were considered significantly altered. Data from 3 independent reticulocyte samples (run in 3 technical replicates) used for metabolomics are shown. *B*, Principal component analysis of metabolomic datasets from reticulocytes and normocytes demonstrates distinct clustering, reflecting the metabolic uniqueness and diversity of reticulocytes. *C*, Bar graph showing the top 25 metabolic pathways enriched in reticulocytes. Key intermediates involved in energy metabolism, nucleotide biosynthesis, and redox homeostasis were more abundant in the reticulocytes. *D*, Heatmap illustrating the relative abundance of representative metabolites in reticulocytes and normocytes. TCA cycle intermediates (citrate, D-glucose-6-phosphate, succinate), nucleotide precursors (uridine, cytidine, NADH), and antioxidants (reduced glutathione) were consistently elevated in reticulocytes, supporting their capacity to manage elevated reactive oxygen species levels associated with active biosynthetic and metabolic processes. Abbreviations: N, normocyte; NADH, reduced nicotinamide adenine dinucleotide; NS, not significant; PC, principal component; R, reticulocyte; TCA, tricarboxylic acid.

Several redox-active metabolites, including reduced glutathione and multiple glutathione conjugates, were significantly enriched in reticulocytes (Supplementary Figure 6), consistent with enhanced antioxidant capacity. Nucleotide precursors (orotate, uridine, cytidine, Adenosine Monophosphate (AMP), Adenosine Diphosphate (ADP), Inosine Monophosphate (IMP) were also elevated (Figure 2*D*), alongside amino acids and intermediates essential for biosynthetic pathways, highlighting the metabolic advantages available to parasites within reticulocytes.

We next compared the metabolomes of parasites developing in reticulocytes versus normocytes. Overall profiles were largely similar (Supplementary Figure 7*A*), with relatively few metabolites differentially regulated. Pathway analysis revealed modest enrichment of energy, lipid, and porphyrin metabolism, with elevated porphyrin intermediates in reticulocyte-derived parasites (Supplementary Figure 7*B*). Nucleotide and glutathione metabolism remained unchanged, suggesting that parasites do not extensively reprogram intrinsic metabolism but instead exploit the metabolically enriched reticulocyte environment.

### Reticulocytes Exhibit Enhanced Oxidative Stress Tolerance Compared to Normocytes

Metabolomic data indicated elevated antioxidant capacity in reticulocytes. Flow cytometry-based ROS quantification using CM-H_2_DCFDA [40, 41] revealed higher basal ROS levels in reticulocytes compared to normocytes (Figure 3*A* and 3*B*). However, upon exposure to TBHP, normocytes accumulated substantially higher ROS, whereas reticulocytes exhibited minimal increases (Figure 3*C*). Western blot analysis further demonstrated elevated levels of ROS-detoxifying enzymes, including superoxide dismutase-1, catalase, and glutathione reductase, in reticulocytes (Figure 3*D*). Together, these findings establish reticulocytes as an erythroid subpopulation with enhanced resilience to oxidative stress, providing a redox-protected intracellular niche for *P*. *falciparum.*

**Figure 3.**
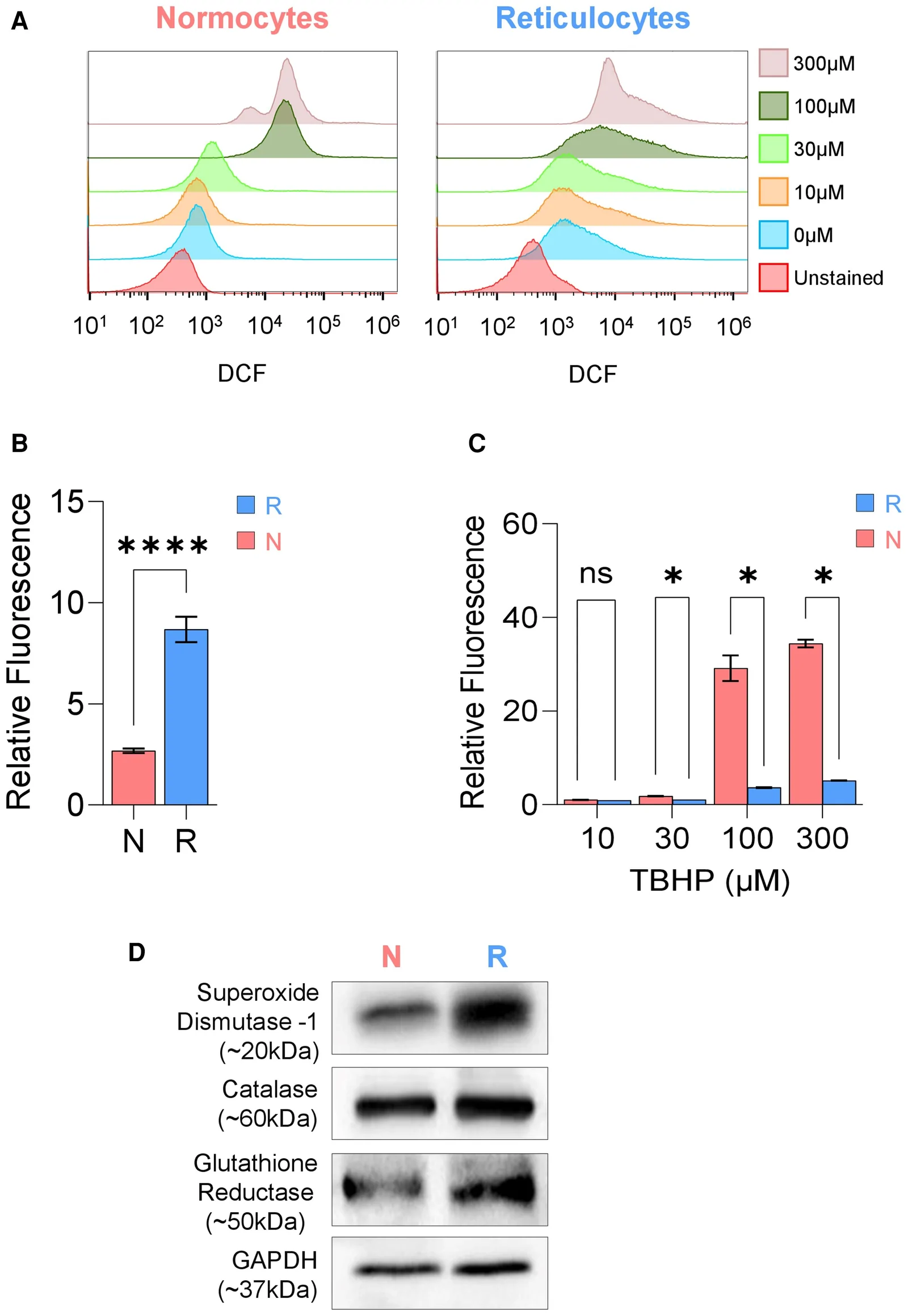
Host reticulocytes offer superior redox attenuation capacity to *Plasmodium falciparum*. *A*, Histograms representing the total reactive oxygen species levels in purified reticulocytes and normocytes quantified through CM-H_2_DCFDA are shown. Flow cytometry was performed after challenging the cells with different concentrations of TBHP. TBHP concentrations used are represented using color codes along with histograms. *B*, Healthy reticulocytes exhibited approximately 3-fold higher baseline ROS levels compared to normocytes, consistent with their higher metabolic and organellar activity. *C*, Reticulocytes and normocytes were exposed to increasing concentrations of TBHP, revealing that reticulocytes tolerate higher oxidative stress. Bar graph shows the distinct oxidative stress responses of reticulocytes and normocytes across TBHP concentrations as fold change in mean fluorescence intensity. Each bar represents the mean ± SD of 3 separate experiments. Statistical significance estimated by multiple unpaired *t* tests; *P ≤ .05 (significant), **P ≤ .01 (significant), ****P ≤ .0001. *D*, Western blot analysis demonstrated elevated levels of the antioxidant enzyme superoxide dismutase-1, catalase, and glutathione reductase in reticulocytes relative to normocytes. GAPDH was used for normalization as the loading control. Abbreviations: CM-H_2_DCFDA, 6-chloromethyl-2′, 7′-dichlorodihydrofluorescein diacetate; GAPDH, glyceraldehyde-3-phosphate dehydrogenase; N, normocyte; ns, not significant; R, reticulocyte; TBHP, tert-butyl hydroperoxide.

### Reticulocyte-Localized Parasites Exhibit Reduced Susceptibility to Artemisinin and Other Redox-Active Antimalarials

Given that artemisinin exerts antimalarial activity through ROS induction, we hypothesized that the superior redox-modulating capacity of reticulocytes could alter parasite drug sensitivity. Consistent with this, parasites cultured in reticulocytes showed markedly reduced sensitivity to artemisinin, dihydroartemisinin, and E64d in 48-hour ring-to-ring assays (Figure 4*A*–*C*), with up to a 10-fold increase in IC_50_ for the artemisinin derivatives. In contrast, pyrimethamine, cycloheximide, and atovaquone showed no host cell-dependent differences (Figure 4*D* and 4*F*), indicating a specific effect on redox-active compounds. E64d inhibits cysteine proteases involved in hemoglobin degradation, which may be better modulated by parasites in the reticulocyte due to abundant amino acids. In contrast, pyrimethamine targets dihydrofolate reductase, atovaquone disrupts mitochondrial electron transport, and cycloheximide inhibits protein synthesis, processes that are independent of host cell redox state. As such, their activity remains unaffected by the distinct environment of reticulocytes, resulting in comparable sensitivity across host cell types.

**Figure 4.**
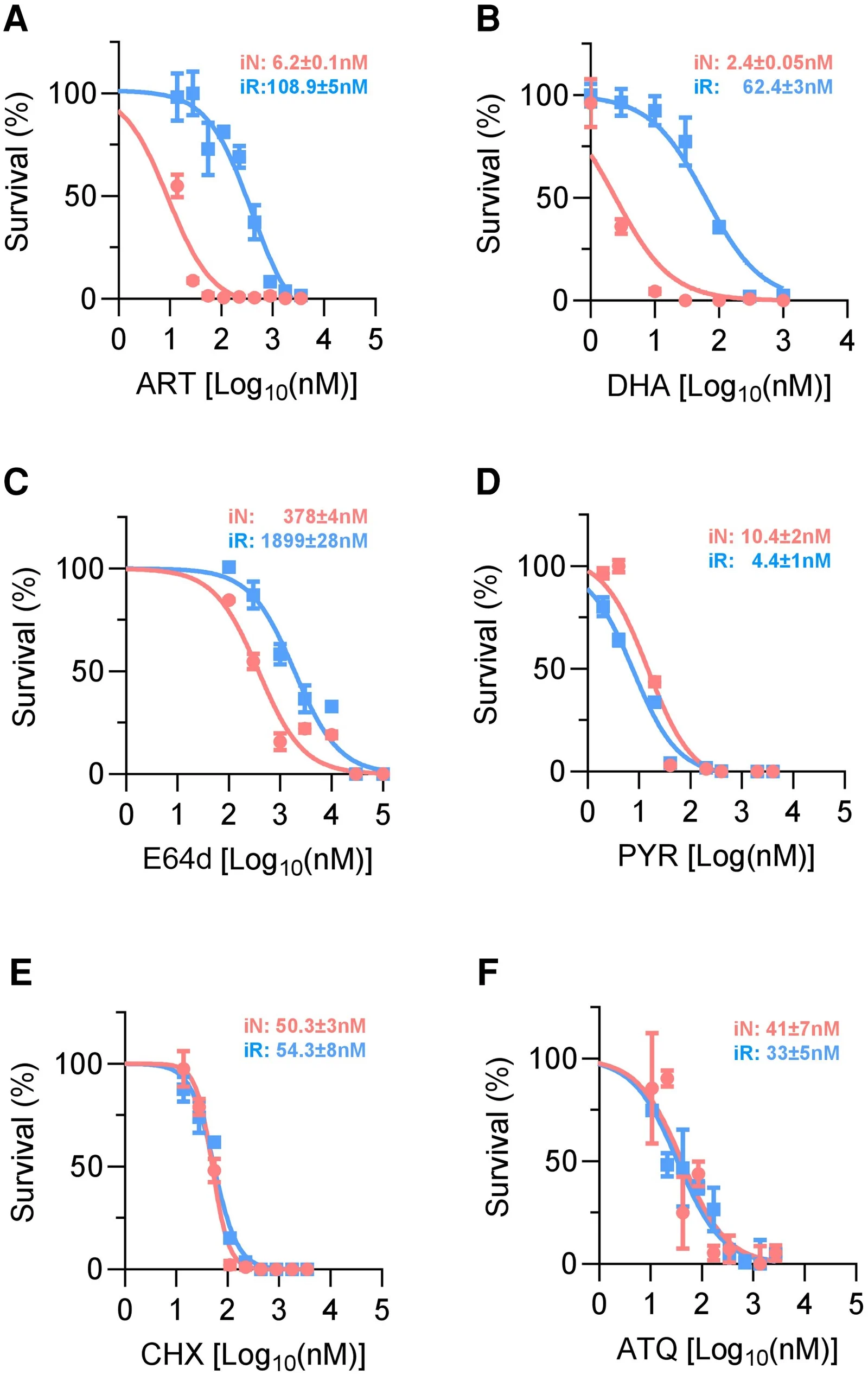
Differential drug tolerance of *Plasmodium falciparum* grown in host reticulocytes. Synchronized schizonts were magnetically purified and allowed to invade either normocytes or reticulocytes. Following ring-stage establishment, cultures were exposed to increasing concentrations of (*A*) ART, (*B*) DHA, (*C*) E64d, (*D*) PYR, (*E*) CHX, and (*F*) ATQ for 48 hours. Parasitemia was quantified by microscopy and validated by flow cytometry to generate dose-response curves. Parasites maintained in reticulocytes displayed markedly reduced sensitivity to ART, DHA, and E64d, while responses to PYR, CHX, and ATQ remained mostly comparable between the 2 host cell types in this 48-hour treatment assay. Estimated IC_50_ (mean ± SD, n = 3) is shown along with the drug response graphs. Abbreviations: ART, artemisinin; ATQ, atovaquone; CHX, cycloheximide; DHA, dihydroartemisinin; IC_50_, 50% maximum inhibitory concentration; iN, infected normocytes; iR, infected reticulocytes; PYR, pyrimethamine.

This phenotype was consistent across multiple donors (Figure 5*A*). Ring survival assays demonstrated similar survival patterns regardless of host cell type (Figure 5*B*), ruling out parasite-intrinsic resistance mechanisms. Additionally, when parasites developed in reticulocytes were introduced to normocytes in the next cycle, they exhibited artemisinin sensitivity comparable to parasites grown continuously in normocytes, highlighting the role of host cell characteristics in modulating drug tolerance (Figure 5*C*). Extended resurgence assays revealed that parasites residing in reticulocytes exhibited enhanced recovery following artemisinin exposure, even at concentrations fully suppressive in normocytes (Figure 5*D*). These findings demonstrate that reticulocytes attenuate drug-induced oxidative stress, enabling parasite survival and resurgence without conferring genetic resistance.

**Figure 5.**
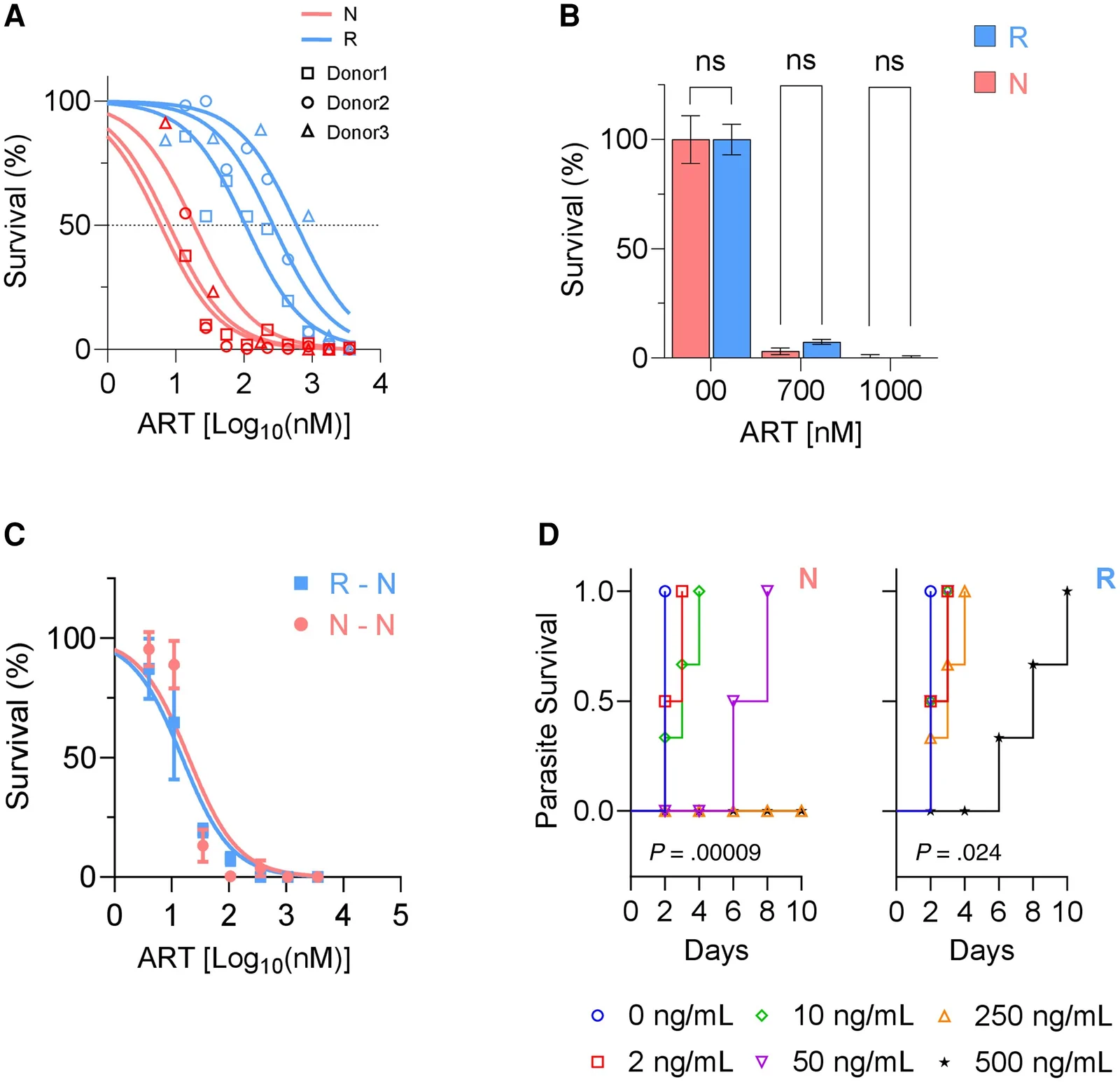
Reticulocyte-mediated tolerance and enhanced resurgence of *Plasmodium falciparum* under artemisinin stress is host-cell dependent. *A*, Dose response curves for artemisinin in parasites grown in reticulocytes versus normocytes from 3 independent donors, showing consistently higher tolerance in reticulocyte-invaded parasites. Data represent median values from 3 technical replicates; parasitemia was quantified by Giemsa-stained smears and validated by flow cytometry. *B*, Ring survival assays at 700 and 1000 nM artemisinin indicate comparable survival between reticulocyte- and normocyte-grown parasites, confirming that reduced susceptibility is due to the host cell environment, rather than parasite-intrinsic changes. *C*, Artemisinin sensitivity of *P. falciparum* parasites was tested after culturing them for 1 cycle in reticulocytes and normocytes followed by seeding on to normocytes. Sensitivity (IC_50_) was measured in the second cycle to assess impact of prior reticulocyte infection on artemisinin sensitivity. *D*, Parasite resurgence after long-term (48 hours) artemisinin exposure is represented using parasite survival plots (Mantel-Cox rank analysis). Parasites were treated with artemisinin for 48 hours, after which the cultures were maintained for 10 days to assess the parasite resurgence across a range of artemisinin concentrations. Mantel-Cox survival curves show rapid, indistinguishable parasite recovery in untreated controls and reticulocyte samples treated with low artemisinin doses (2, 10, 50 ng/mL), resulting in overlapping survival lines that diverge at higher concentrations. *P* values are shown in the graphs along with the host cell type information. Abbreviations: ART, artemisinin; IC_50_, 50% maximum inhibitory concentration; N, normocyte; ns, not significant; R, reticulocyte.

Collectively, these results identify a previously unrecognized host cell-attenuation mechanism of artemisinin tolerance. The reticulocyte cytosolic environment provides a biochemical sanctuary that protects *P. falciparum* from ROS-driven killing, fundamentally influencing drug efficacy and offering a mechanistic explanation for variable treatment outcomes in reticulocyte-rich infections.

## DISCUSSION

*P. falciparum* has long been regarded as an exceptional parasite for its ability to invade erythrocytes across all maturation states, yet the functional consequences of this breadth remain incompletely understood. Here, we demonstrate that *P. falciparum* displays a quantifiable preference for reticulocytes and that this preference influences parasite physiology, from invasion efficiency and intraerythrocytic growth dynamics to redox physiology and drug tolerance. Our findings uncover reticulocytes not simply as permissive host cells but as a metabolically privileged niche exploited by the parasite, with direct consequences for clinical manifestation and treatment response. Importantly, this phenotype reflects host cell-dependent drug tolerance rather than parasite-intrinsic resistance, highlighting a nongenetic mechanism of artemisinin tolerance.

We demonstrate that *P. falciparum* invades reticulocytes at higher rates than normocytes across multiple parasite strains and erythrocyte sources. This establishes that the erythrocyte age, not necessarily hemoglobin composition [42], as the dominant determinant. These observations expand findings from clinical isolates, providing quantitative evidence that human reticulocytes constitute a preferred entry route [7]. The biological significance of this preference extends beyond merozoite invasion: in settings of hemolytic anemia or marrow stress, where reticulocytosis is pronounced, parasites would gain a growth advantage, offering a mechanistic explanation for the well-established correlation between anemia and increased parasitemia [14, 15]. This also aligns with recent observations of disproportionate CD71^+^ RBC infection in severe malaria [43], suggesting that reticulocyte targeting could serve as a biomarker of pathogenic infections.

Once inside reticulocytes, *P. falciparum* undergoes accelerated development without altering replication rate, indicating that the host cell environment modulates growth kinetics. The metabolite-rich landscape of reticulocytes, characterized by elevated glycolytic flux, robust glutathione metabolism, active tricarboxylic acid intermediates, and sustained biosynthetic capacity, appears to support faster parasite maturation. These data integrate well with recent reports of heightened gametocyte conversion in bone marrow reticulocytes [13–16]. The broader implication is that the bone marrow niche, enriched in metabolically active reticulocytes, may serve as a cryptic site not only for sexual conversion but also for accelerated asexual amplification.

A major conceptual advance from this study is the demonstration that the redox environment of reticulocytes can contribute to parasite drug tolerance. Parasites developing in CD71^+^ cells show reduced sensitivity to artemisinin and related compounds. Recovery assays confirm that parasites in reticulocytes can withstand and rebound from drug exposure far more efficiently than those in normocytes. Mechanistically, this can be attributed to the inherently reductive environment of reticulocytes. By acquiring reticulocyte-derived ROS-quenching machinery, both inside the parasites and outside the parasitophorous vacuole, parasites weaken artemisinin-induced oxidative bursts.

Current frameworks of artemisinin resistance focus primarily on parasite-encoded factors such as *kelch13* mutations [44, 45], altered protein damage responses [46], or quiescence pathways [47–49]. Our data suggest that redox physiology of the infected host cell can be influential in shaping drug response. Reticulocytes, rich in antioxidant defenses, may help attenuate the artemisinin-induced oxidative and protein damage, suggesting that the host environment is not merely permissive but potentially providing a protective host cell milieu in which the parasites can quench artemisinin damage. Such effects become particularly relevant in niches enriched for reticulocytes, such as the bone marrow. Individual components such as reduced glutathione (GSH), glutathione synthase, and peroxiredoxins have already been proven to influence the ability of *P. falciparum* parasites to tolerate artemisinin [24–26, 32, 33]. Upregulation of such components in the reticulocytes may help explain phenomena such as delayed parasite clearance in anemia, variability in drug response across hematologic states, and potential persistence of parasites within extracirculatory reservoirs after treatment.

Notably, parasites developing inside reticulocytes exhibited increased tolerance against E64d, which kills parasites primarily by disrupting hemoglobin degradation. This finding aligns with established advantages provided by reticulocytes to parasite mutants such as those lacking critical enzymes needed for hemoglobin digestion, which survive in reticulocytes [28]. The upregulation of essential metabolic intermediates in reticulocytes likely enables parasites to bypass hemoglobin degradation through alternative nutrient acquisition pathways. This metabolic flexibility may partly explain the observed artemisinin tolerance, as reduced hemoglobin digestion restricts free heme essential for the activation of artemisinin's endoperoxide bridge, a mechanism which needs further exploration.

Together, these findings position reticulocytes as a distinct microenvironment that shapes core aspects of *P. falciparum* pathophysiology. These insights carry clinical relevance: therapies targeting redox exchange, host antioxidant import, or reticulocyte-specific interactions may enhance antimalarial efficacy, and stratifying patients by hematologic status may refine treatment expectations and protocols. Beyond *P. falciparum*, these findings have direct implications for reticulocyte-restricted species such as *P. vivax* and *P. ovale*, suggesting that host redox attenuation may contribute to their distinctive growth characteristics and drug-response profiles. Future work will be required to elucidate the molecular machinery involved in host-parasite antioxidant transfer and to define how marrow reticulocyte niches contribute to treatment-refractory infections.

## CONCLUSION

Our study reveals that human reticulocytes constitute a metabolically privileged and redox-enriched intracellular niche that impacts *P. falciparum* biology—promoting enhanced invasion, accelerating the asexual cycle, and conferring elevated tolerance to redox-active antimalarials. By leveraging purified CD71^+^ reticulocytes, an exceptionally scarce, short-lived, and experimentally challenging erythroid population, we demonstrate that parasites exploit the enriched antioxidant reservoir of immature erythrocytes, uncovering a host-cell–driven mechanism of drug protection that operates independently of parasite genotype. These findings position erythroid maturation state as a determinant of parasite behavior across hematopoietic niches, providing a unifying explanation for bone-marrow sequestration, anemia-associated hyperparasitemia, and interindividual variability in treatment response. Looking forward, dissecting the specific host-parasite metabolic and redox exchanges that occur uniquely within reticulocytes, mapping how these pathways vary across human physiological states, and testing strategies that disrupt host-derived antioxidant buffering represent important next steps. Such approaches may open new therapeutic avenues for overcoming artemisinin tolerance and improving antimalarial efficacy in the clinical setting.

## Supplementary Material

jiag290_Supplementary_Data

## Supplementary Data

Supplementary materials are available at *The Journal of Infectious Diseases* online (http://jid.oxfordjournals.org/). Supplementary materials consist of data provided by the author that are published to benefit the reader. The posted materials are not copyedited. The contents of all supplementary data are the sole responsibility of the authors. Questions or messages regarding errors should be addressed to the author.
