# Supplementary material for: Host Reticulocyte Redox Attenuation Creates a Protective Niche for Artemisinin Tolerance in Plasmodium falciparum
Source: J Infect Dis. 2026 Jun 16;234(4):e940–51. doi: 10.1093/infdis/jiag290 (PMC13668372; doi:10.1093/infdis/jiag290)
Supplement: Supplementary file 1 [file jiag290_supplementary_data.zip › Supplementary Methods_01052026_CD_Cleaned up.pdf]

Supplementary Methods

Metabolome profiling

Reticulocytes and normocytes isolated through magnetic selection was used for metabolomic profiling. Purity of samples was confirmed using flow cytometry (using Thiazole Orange) and microscopy (using New Methylene Blue), which was above 95% (Supplementary Figure 1). After counting the cells using a hemacytometer, 10^8 cells were subjected to metabolite extraction. For this, cells were lysed by flash- freezing and thawing followed by addition of 100μL cold methanol at 4°C for 15 min. Next, cells/membranes/debris were pelleted down by high-speed centrifugation (21,000 g, 15 min, 4°C), supernatants were collected, dried in a SpeedVac, and stored at –20 °C until analysis.

For performing metabolomics on parasite fraction, pure magnet-isolated reticulocytes and normocytes were infected with *P. falciparum* and allowed to propagate till trophozoite stage (~ 30hpi). After counting the cells using a hemacytometer, 10^8 infected cells were collected and lyzed by treatment with 0.2% saponin, and the parasite pellet was washed thrice in ice-cold PBS. Thereafter, methanol extraction was conducted for metabolite isolation as described above.

Metabolites were separated using *Vanquish UHPLC* system equipped with a *Waters* reverse-phase (RP) column (2.1 x 150 mm, particle size 1.8 um). Samples were separated by maintaining 45°C temperature under still air condition. 0.1% formic acid in water used as mobile phase A, and 0.1% formic acid in methanol used as mobile phase B. Mass spectroscopy was performed using *Eclipse Orbitrap* mass spectrometer with heated electron spray ionization (H-ESI) source. Spray voltage was maintained in static mode at 3400 V for positive and 2800 V for negative ion detection. The sheath gas, auxiliary gas, and sweep gas flows were maintained at 40, 5, and 0 arbitrary units, respectively. The capillary temperature was maintained at 300°C, and the vaporizer temperature was set at 400°C. Key acquisition parameters included a resolution setting of 120,000, 1 microscan per scan, with a scan range of m/z 100-1000.

For data-dependent acquisition (DDA), the instrument was programmed for full MS/dd-MS2 scan with polarity switching in both positive and negative modes. The MS scans were acquired using a resolution of 60,000, 1 microscan, and a scan range of m/z 70-1000. MS/MS (dd-MS2) fragmentation was performed in the Orbitrap using higher-energy collisional

dissociation (HCD) with stepped collision energies of 20%, 35%, and 50%. The MS2 scans were acquired at a resolution of 15,000, with an isolation window of 1.5 m/z.

Compounds were identified using *mzCloud* (ddMS2) and *ChemSpider*. Statistical significance was estimated using t-test. Annotated features were selected for the analysis. Multivariate analysis, hierarchical clustering heatmaps, and pathway enrichment analysis were conducted in *MetaboAnalyst* 6.0. Volcano plots were generated in R. Complete metabolomics data is presented as **Supplemental Dataset. 1.**

Western Blotting

For Western blotting, healthy reticulocytes and normocytes lysates were loaded based on cell number for SDS-PAGE and transferred to a nitrocellulose membraneand probed with anti-SOD1 (Proteintech, #10269-1-AP; 1:5000) anti-catalase (Cell Signaling Technology, #12980; 1:4000), anti-GSR (Invitrogen #MA53478; 1:5000), anti-GAPDH (Invitrogen, #MA5-15738; 1:10,000) antibodies. Goat anti-rabbit HRP-conjugated secondary antibody (Invitrogen, #31460; 1:10,000) and Goat anti-mouse HRP-conjugated secondary antibody (Invitrogen, #62-6520; 1:8,000) were used for ECL-based detection on a iBright™ CL1500 Imaging System (Thermofisher Scientific).
