# Supplementary material for: Host Reticulocyte Redox Attenuation Creates a Protective Niche for Artemisinin Tolerance in Plasmodium falciparum
Source: J Infect Dis. 2026 Jun 16;234(4):e940–51. doi: 10.1093/infdis/jiag290 (PMC13668372; doi:10.1093/infdis/jiag290)
Supplement: Supplementary file 1 [file jiag290_supplementary_data.zip › SUPPLEMENTARY.pdf]

Supplementary Figure. 1

A

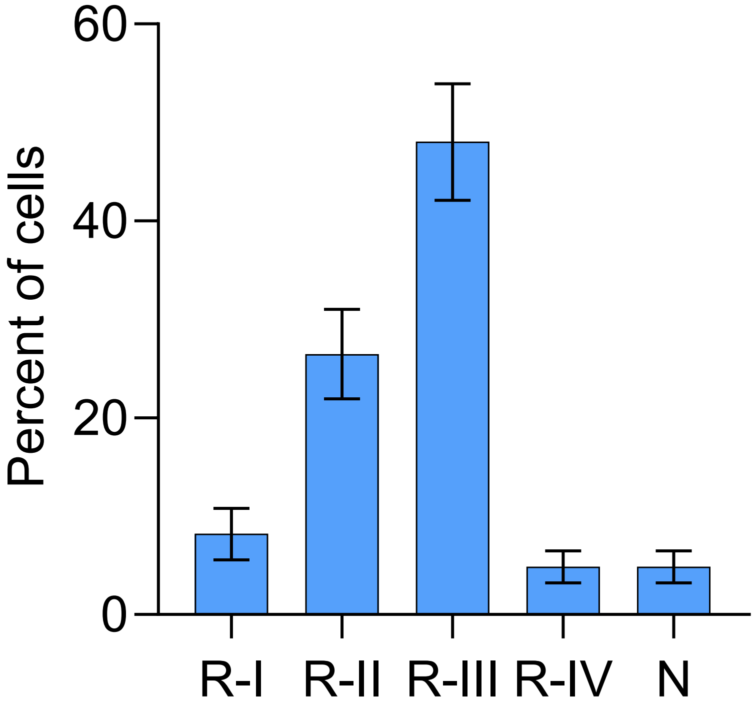

B

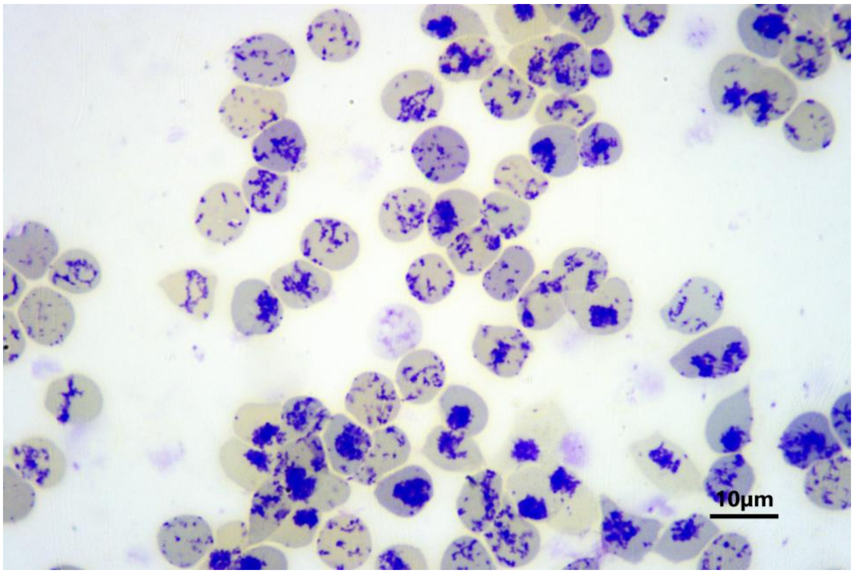

Supplementary Figure. 2

A

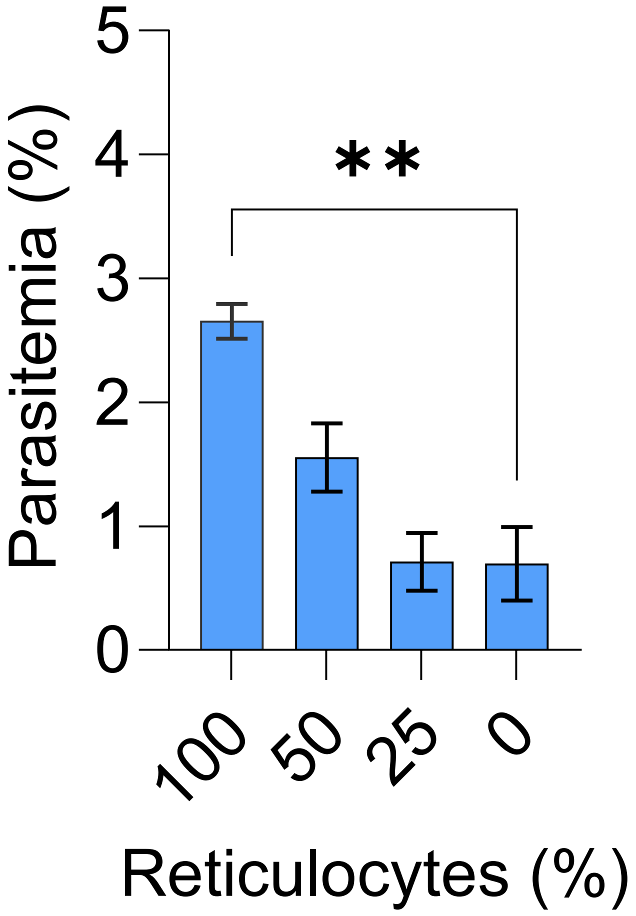

Supplementary Figure. 3

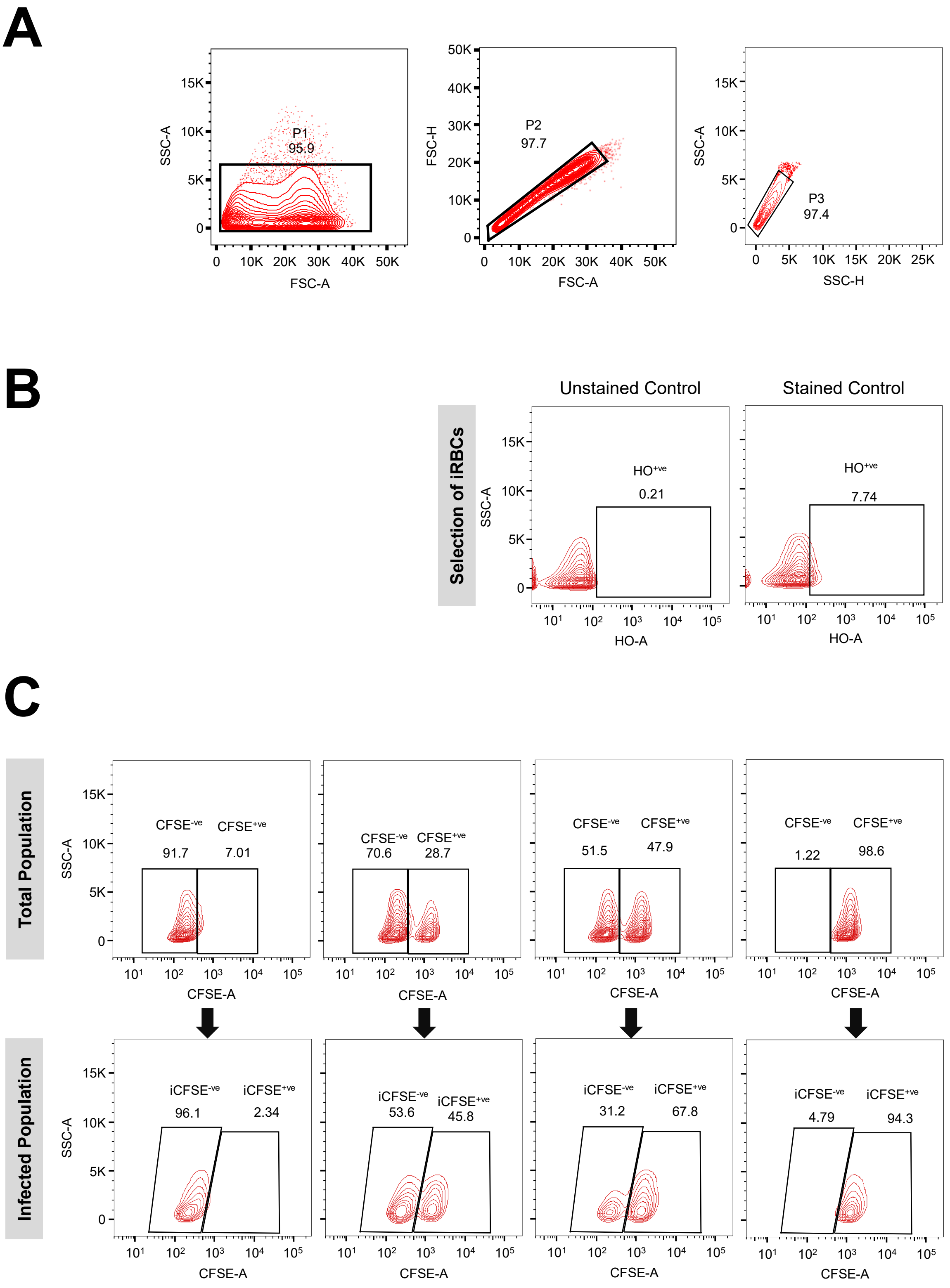

Supplementary Figure. 4

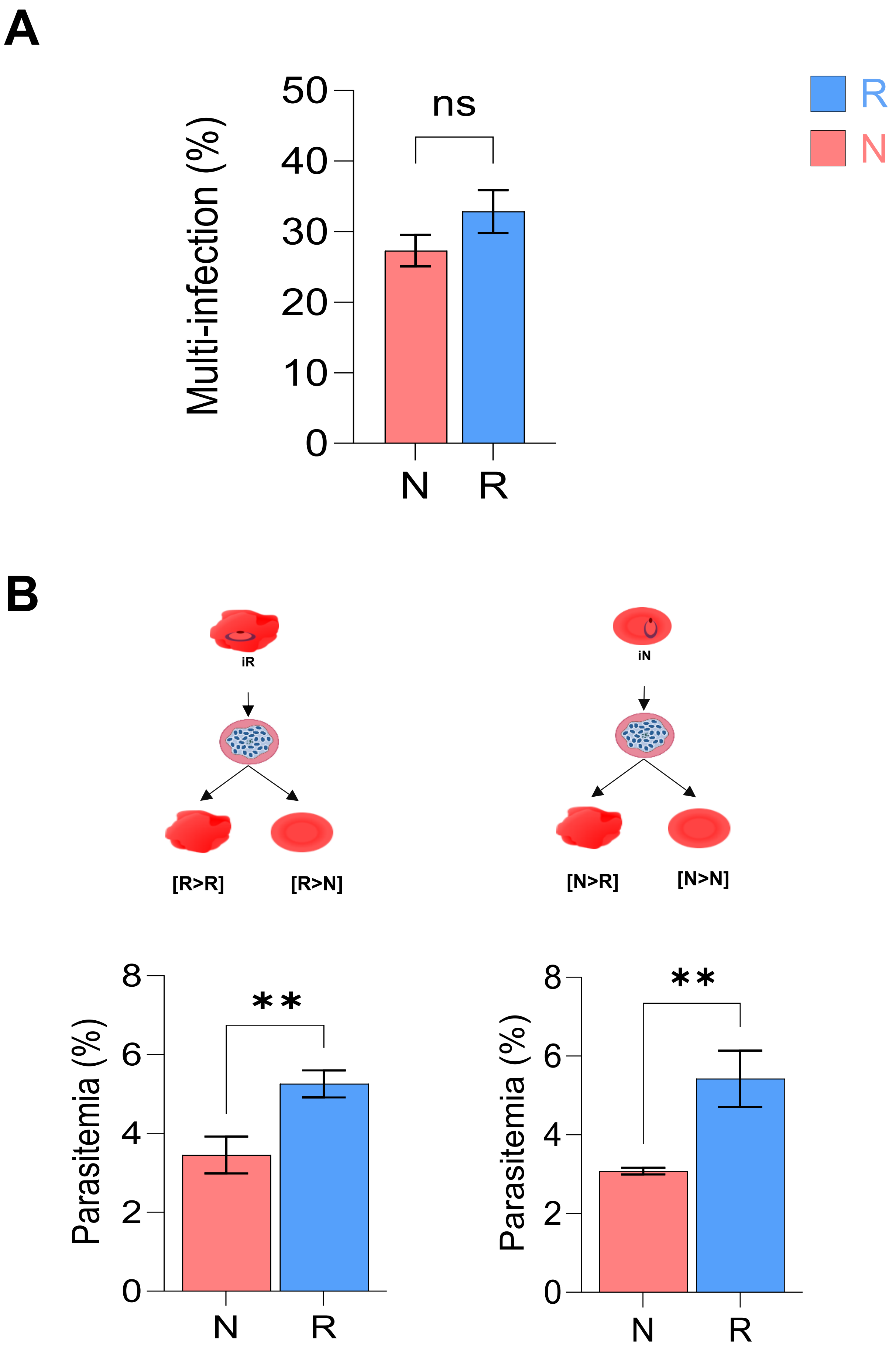

Supplementary Figure. 5

A

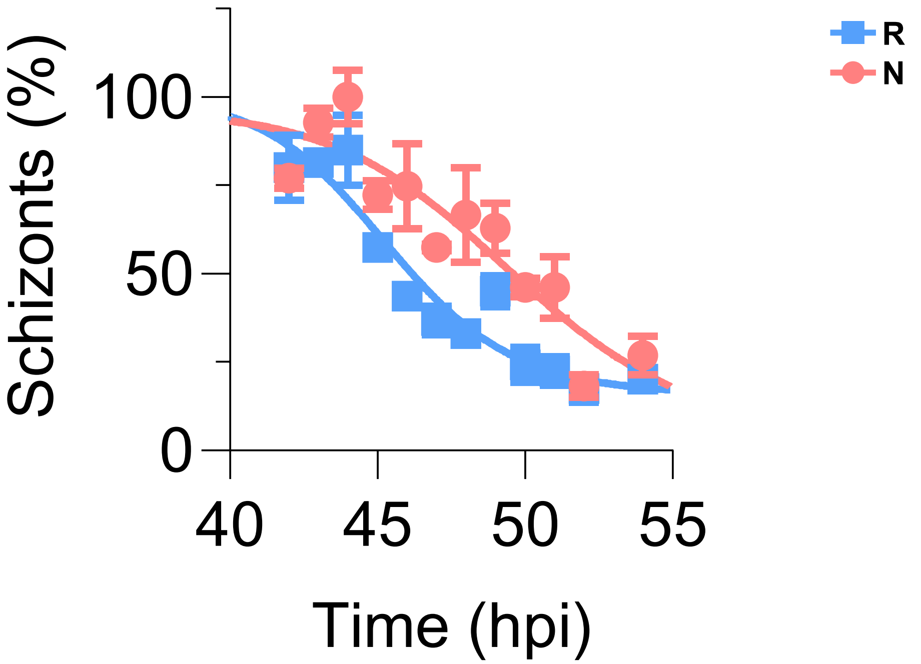

B

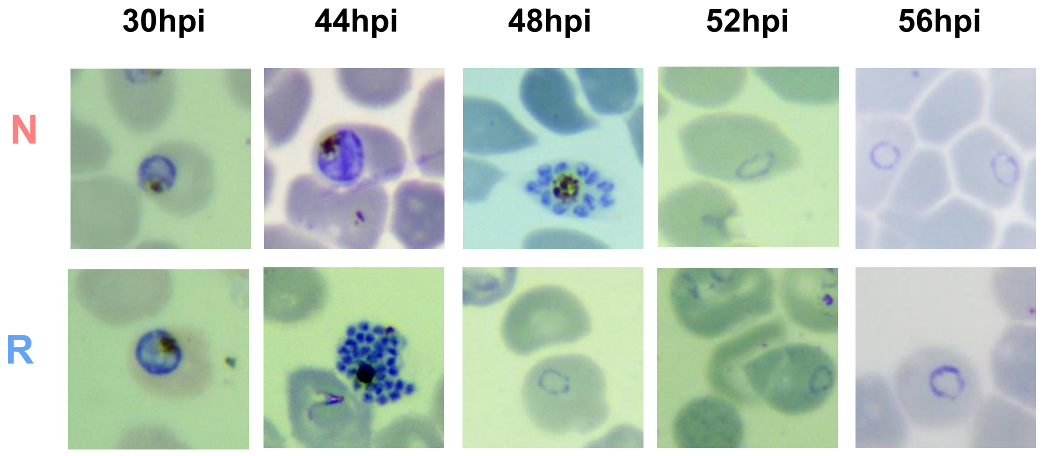

Supplementary Figure. 6

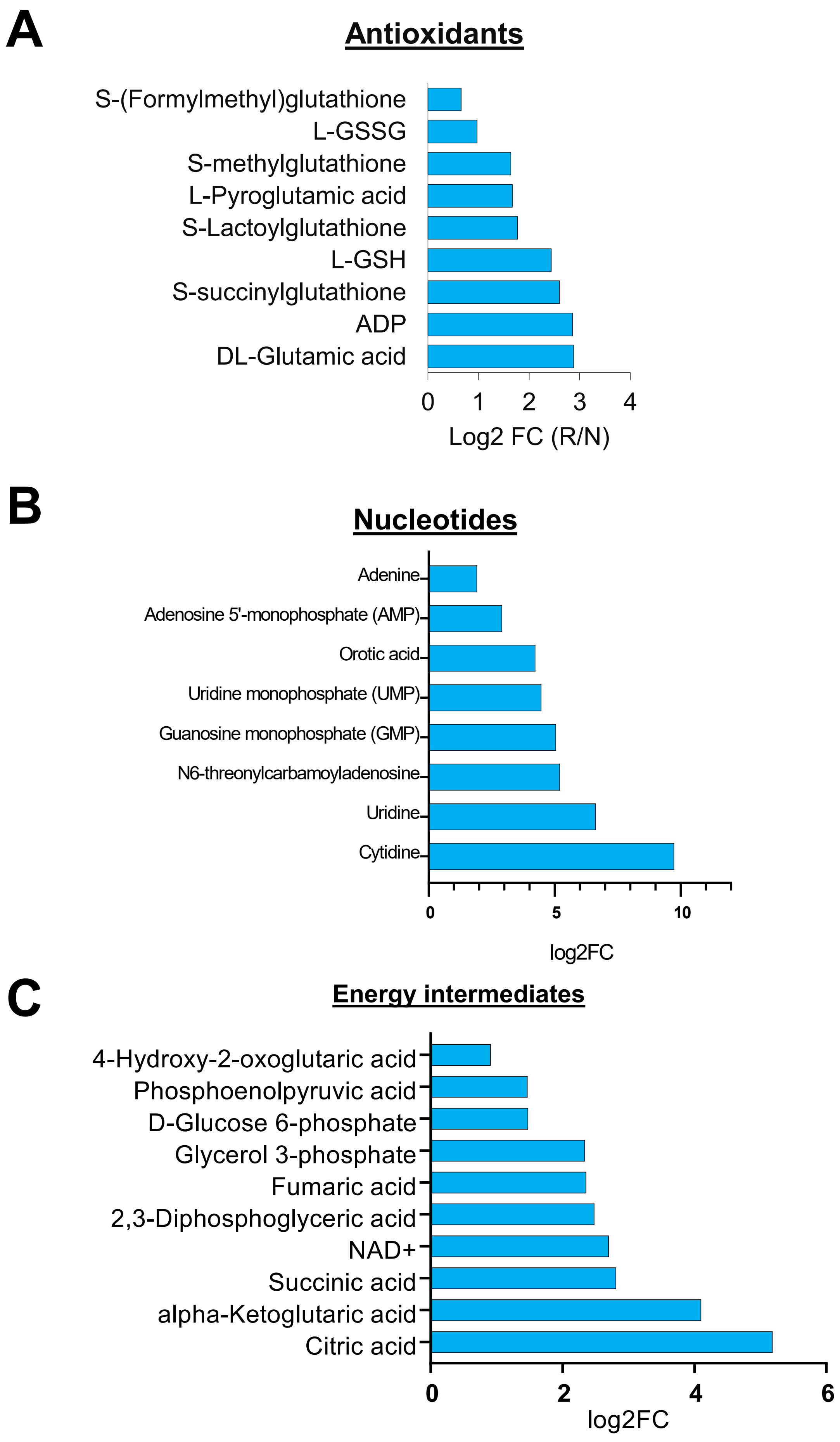

Supplementary Figure. 7

A

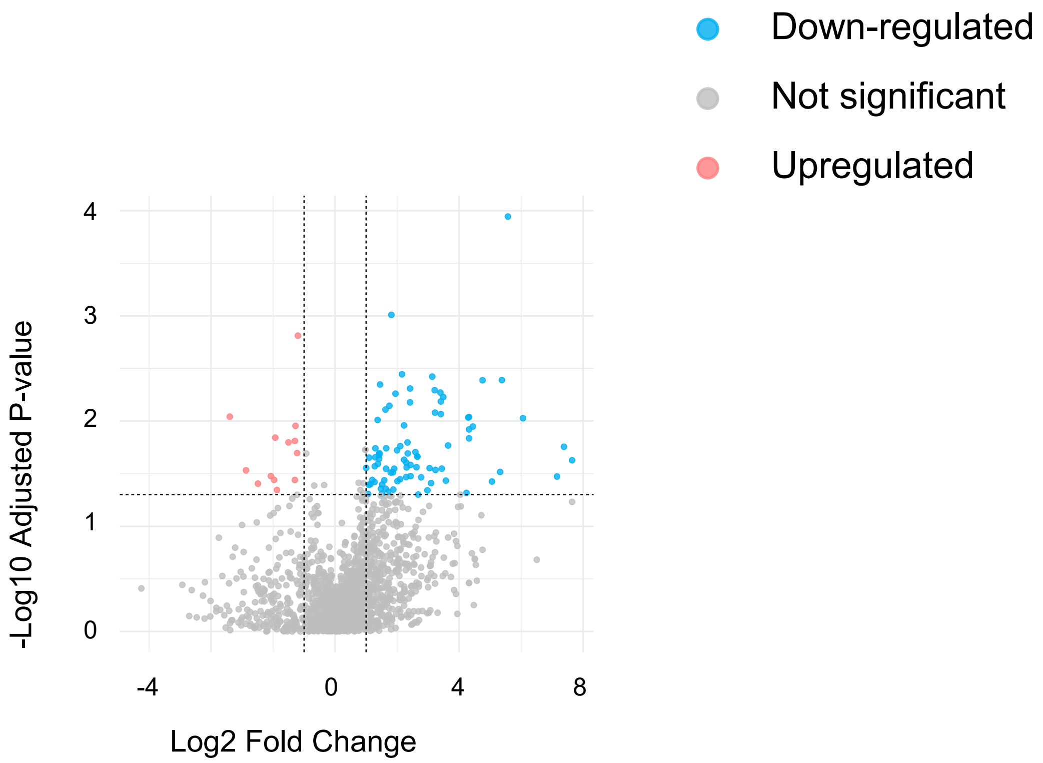

B

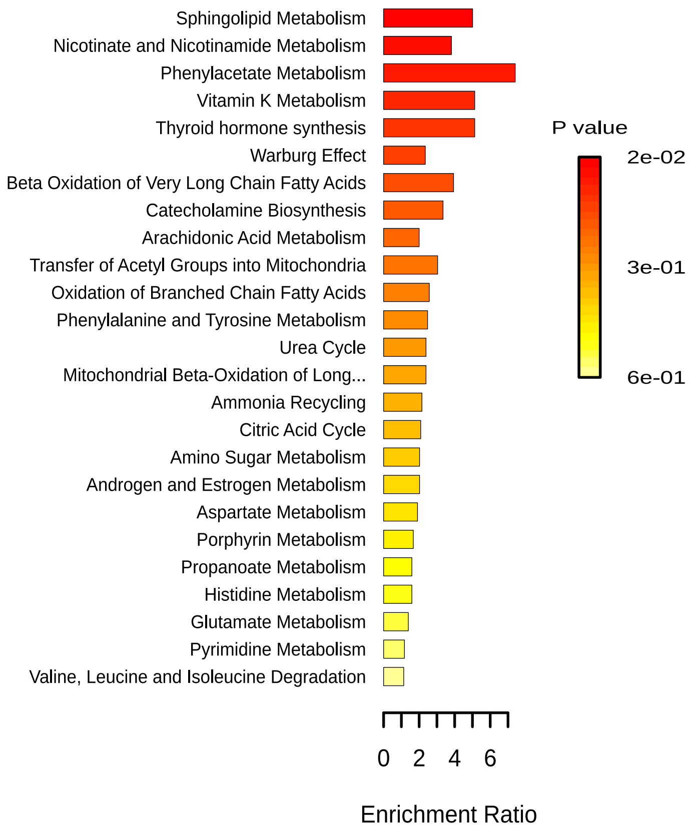

Supplementary Figure. 8

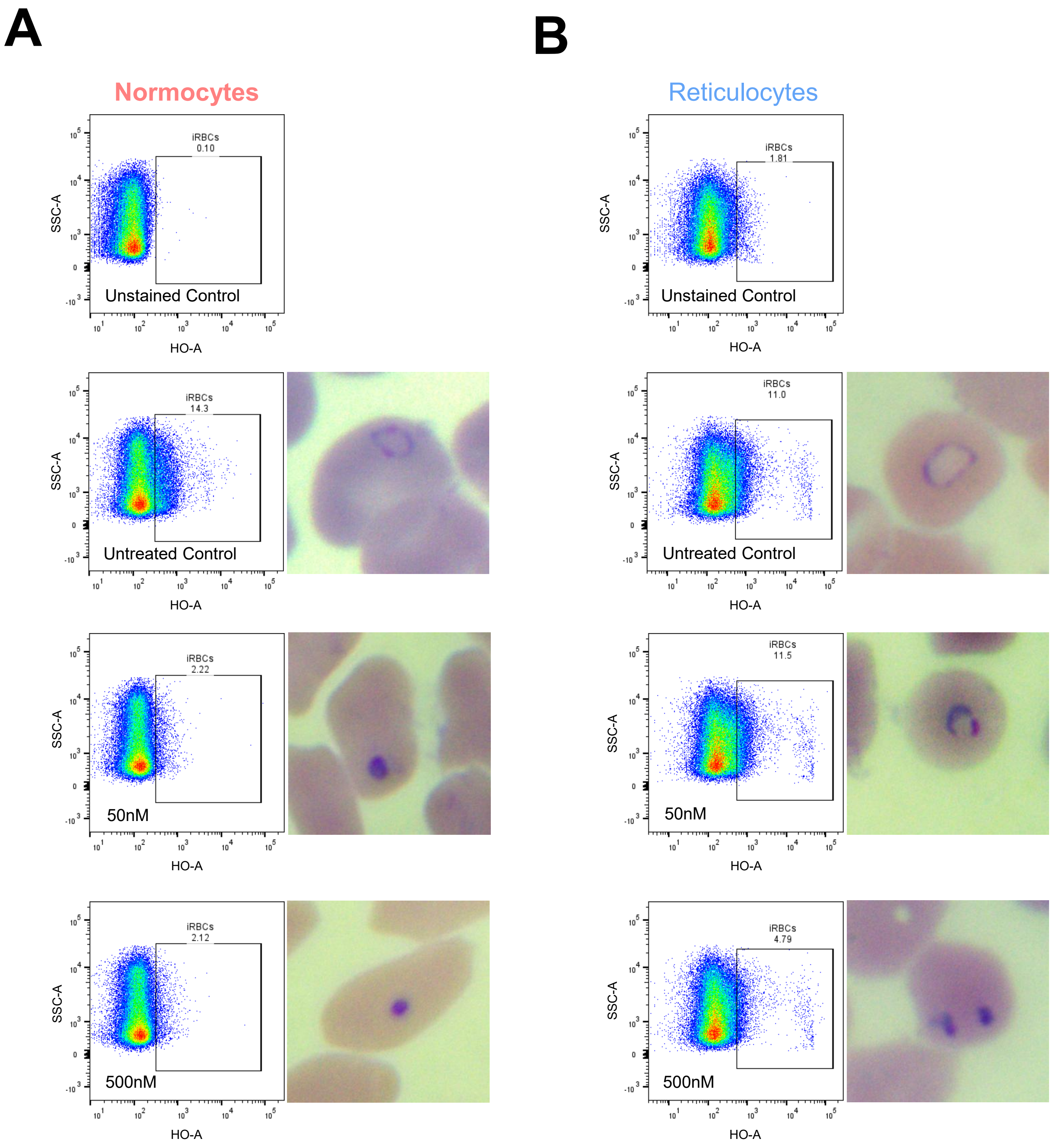

Supplementary Figure Legends

Figure. S1: Relative abundance of reticulocyte stages (RI-RIV) in MACS- purified samples. (A) Bar graph representing the relative abundance of reticulocyte stages (RI-RIV) isolated using MACS, quantified from the microscopy images of new methylene blue (NMB) stained samples. Data represents the pooled results of five independent biological samples. (B) Microscopy image showing NMB staining pattern of reticulocytes enriched by MACS, confirming higher abundance of R-III.

Figure. S2: Total parasitemia determination at different ratios of reticulocytes to normocytes. At higher reticulocytemia, significant increase in ring-stage parasites was observed. Error bar represents the Standard Deviation (SD) of three independent replicates (n=3). Experiments were repeated thrice to validate the reproducibility, p-value were assessed by unpaired t-test. ** indicates $p < 0.01$.

Figure. S3: Flow cytometry and gating approach used to determine reticulocyte preference by mixing normocytes ($CFSE^-$) with varying amounts of pre-stained reticulocytes ($CFSE^+$). (A) Gating strategy used to select erythrocyte population of each sample for downstream analysis. (B) Gating strategy to differentiate ring stage *Plasmodium* infected (HO^+) from uninfected erythrocytes (HO^-), based on unstained and Hoechst (HO) stained controls. (C) Gating to segregate Reticulocytes ($CFSE^+$) and normocytes ($CFSE^-$) within total population (Upper panel) and *P. falciparum* infected sub-population (Lower panel).

Figure. S4: (A) The extent of multiple infection in normocytes vs reticulocytes based on appearance of ring stage parasites. Infected erythrocytes with more than one ring were counted as multi-infections. In each of three independent experiments, 100 infected erythrocytes per sample were counted. **(B)** Possible changes in invasion preferences was determined by culturing parasites in reticulocytes (left panel) and normocytes (right panel) till late schizont stages (~ 44 hpi). These were purified to infect fresh reticulocytes and normocytes and parasitemia was estimated as mean ± SD (n=3). Statistical significance was estimated by unpaired t-test (ns = $p > 0.05$; * = $p \leq 0.05$; ** = $p \leq 0.01$).

Figure. S5: Demonstration of accelerated intraerythrocytic cycle within reticulocytes was estimated by schizont egress analysis. (A) Time point at which 50% schizonts egressed was compared between infected reticulocytes and normocytes by non-linear regression. Data presented are the mean ± SD (n=3). (B) Microscopic images representing parasite progression over different time points was performed to match the findings.

Figure. S6: Bar graphs representing the relative abundance of individual metabolites involved in the **(A)** glutathione metabolism, **(B)** nucleic acid metabolism, and **(C)** energy intermediates.

Figure. S7: Metabolome profiling of parasite fraction of infected reticulocytes and infected normocytes. **(A)** Volcano plot representing the metabolome comparison of parasites grown in reticulocytes vs normocytes as host cells. Despite major changes in host cell metabolome within which parasites reside, parasite metabolism appeared mostly comparable. Metabolites with $-\log_{10}$ (p-value) greater than 1.3 and $\log_2$ fold change >1 were selected for downstream analysis. **(B)** Bar graph representing the top 25 metabolic pathways enriched in parasites of infected reticulocytes.

Figure. S8: Gating strategy for segregating uninfected and infected normocytes (A) and reticulocytes (B) through flow cytometry. Infected and uninfected cells were segregated based on the staining pattern of DNA stain Hoechst (HO). Corresponding images of Giemsa stained cells highlight parasite phenotypes along with the flow plots.
